# An exploration of the experiences of professionals supporting patients approaching the end of life in medicines management at home. A qualitative study

**DOI:** 10.1186/s12904-020-0537-z

**Published:** 2020-05-11

**Authors:** Eleanor Wilson, Glenys Caswell, Asam Latif, Claire Anderson, Christina Faull, Kristian Pollock

**Affiliations:** 1 School of Health Sciences, University of Nottingham, Nottingham, UK; 2 Nottingham Centre for the Advancement of Research in End of life care (NCARE), B302 School of Health Sciences, Medical School, Queen’s Medical Centre, University of Nottingham, Nottingham, NG7 2UH UK; 3 School of Pharmacy, University of Nottingham, Nottingham, UK; 4 LOROS Hospice, Leicester, UK

**Keywords:** Managing medication, Healthcare professionals, End of life care, Patients, Qualitative research, Pharmacy, Dose administration aids

## Abstract

**Background:**

The management of medicines towards the end of life can place increasing burdens and responsibilities on patients and families. This has received little attention yet it can be a source of great difficulty and distress patients and families. Dose administration aids can be useful for some patients but there is no evidence for their wide spread use or the implications for their use as patients become increasing unwell. The study aimed to explore how healthcare professionals describe the support they provide for patients to manage medications at home at end of life.

**Methods:**

Qualitative interview study with thematic analysis. Participants were a purposive sample of 40 community healthcare professionals (including GPs, pharmacists, and specialist palliative care and community nurses) from across two English counties.

**Results:**

Healthcare professionals reported a variety of ways in which they tried to support patients to take medications as prescribed. While the paper presents some solutions and strategies reported by professional respondents it was clear from both professional and patient/family caregiver accounts in the wider study that rather few professionals provided this kind of support. Standard solutions offered included: rationalising the number of medications; providing different formulations; explaining what medications were for and how best to take them. Dose administration aids were also regularly provided, and while useful for some, they posed a number of practical difficulties for palliative care. More challenging circumstances such as substance misuse and memory loss required more innovative strategies such as supporting ways to record medication taking; balancing restricted access to controlled drugs and appropriate pain management and supporting patient choice in medication use.

**Conclusions:**

The burdens and responsibilities of managing medicines at home for patients approaching the end of life has not been widely recognised or understood. This paper considers some of the strategies reported by professionals in the study, and points to the great potential for a more widely proactive stance in supporting patients and family carers to understand and take their medicines effectively. By adopting tailored, and sometimes, ‘outside the box’ thinking professionals can identify immediate, simple solutions to the problems patients and families experience with managing medicines.

## Background

Professional focus on medicine prescribing and adherence has sometimes been accompanied by a lack of awareness of the concerns which patients frequently have about their medicines, and the burden and practical difficulties involved in taking them [1]. Notenboom et al. [2] demonstrate some of the pragmatic challenges patients face when charged with managing medications in the home. Study participants cited 211 problems with using oral prescription medications, with 95% of participants identifying at least one issue. Participants reported employing 184 strategies to manage these practical problems. Seventeen percent of participants experienced a medication issue that led to clinical deterioration. The participants in the study identified a range of issues around reading and understanding the instructions for use (also see [3]), difficulties handling packaging, and physical issues around taking medicines [2].

**Fig. 1:**
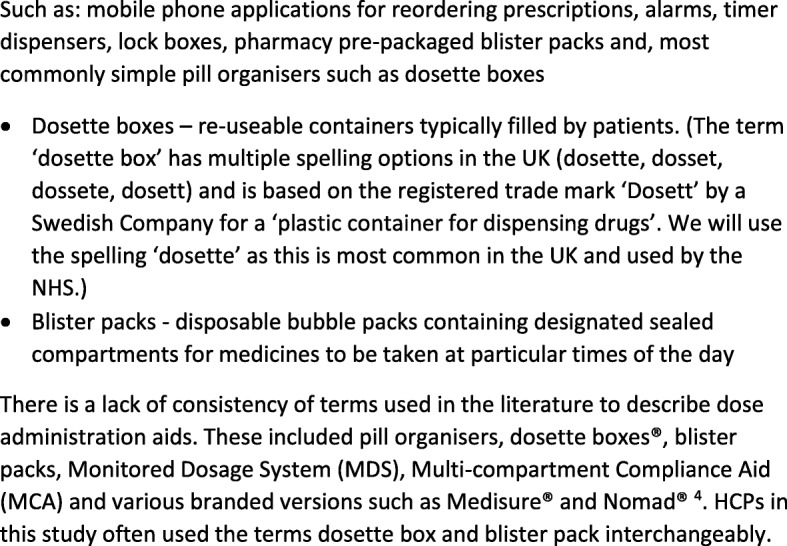
Dose administration aids

In the UK guidelines have been developed for professionals that focus on involving patients and their family members in decisions about medications and prescribing [4]. Alsaeed et al.’s [5] literature review highlights a number of areas in which healthcare professionals (HCPs) could help to improve patients’ medicines management at home. While the focus of the review was on patients with dementia and their caregivers, many of their recommendations are more broadly applicable. Good communication, between patient and family caregiver (FCG), between families and HCPs, and between HCPs is fundamental to identifying and managing any issues with medication [6]. Providing clear information about medications and undertaking regular reviews were also central elements that promote and support medicines management [5] (also see [1]). There are a number of dose administration aids [see Fig. 1] and technologies available for patients and FCGs to support management and administration. Multi-compartment compliance aids can be extremely useful for some but do have a number of limitations [7, 8] and there is currently insufficient evidence to support their widespread use [4, 9, 10].

**Fig. 2:**
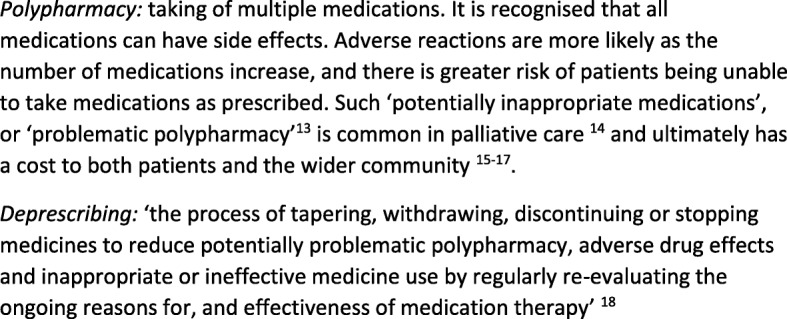
Polypharmacy and deprescribing

Research into older age, including the impact of dementia, is contributing to our knowledge about the role patients and FCGs must take on in order to manage medications [1, 2, 11, 12]. This work has identified that patients often have to cope with complex regimes and require considerable support from FCGs. Managing medications when someone is seriously ill and dying at home can generate additional issues. However, little attention has been paid to the context of palliative and end of life care. As patients become increasingly unwell, they often require frequent and irregular changes to their medication regimes. This may include stopping some long-term preventative medicines such as statins, but increasing others such as those for pain and nausea (see Figure 2 on Polypharmacy and deprescribing [13–18]). Patients may begin to struggle with swallowing standard tablets and medications may be dispensed in other forms such as patches, liquids or sublingually. There is increasing evidence that family caregivers experience a number issues in adjusting doses and responding to rapid drug changes to control difficult and distressing symptoms in the period leading up to the patient’s death [19].

## Methods

### Study design

Qualitative study underpinned by a social constructionist perspective involving semi-structured in-depth interviews. Interviews were situated accounts of HCPs’ perspectives and understanding. In their different roles HCPs engage with patients and families in a variety of ways, in clinics, pharmacy/community settings and in their own homes allowing them to identify practical day-to-day issues and offer support. This paper draws on a set of findings from a wider NIHR funded UK study to explore the management of medications for patients who are approaching end of life. We report on how HCPs described the way in which they supported patients and families to manage medications in their homes. Patient and family perspectives will be reported elsewhere.

### Setting

The study is set in two English counties. Both are diverse in levels of affluence, age, ethnicity and population density, with both rural and urban areas. In both regions there are generalist and specialist teams providing palliative and end of life care.

### Participants

Participants included 40 community professionals from a range of roles, including: General practitioners (GP), specialist palliative care nurses, community nurses and both GP- and community-based pharmacists (see Table 1). Participants were recruited using purposive and snowballing techniques across the two counties via GP practices, specialist palliative care services and via email to a number of key contacts who were asked to distribute the information to their networks.

**Table 1:** Participants by profession

Role	Number of participants
Specialist palliative care nurses^a^	15
Community nurses^b^	12
Consultants (palliative medicine, gerontology)	2
Pharmacists	4
GPs	7
**Total**	**40**

^*a*^*Specialist palliative care nurses* – those working at a Band 6 or 7 level with additional training in palliative care. These may include: *Clinical Nurse Specialists*/*Hospice at home nurses* – these nurses provide advice and support to patients and families as well as having expertise in the management of symptoms. They may be assigned to work in patients’ homes or with care homes. *Macmillan Nurses* – some specialist palliative care nurses are badged under the brand of ‘Macmillan Cancer Support’, a national cancer charity. These nurses focus on providing advice for the management of complex symptoms and/or psychological distress

^b^This is an overarching group of nurses working in the community including District nurses, Community Matrons and Clinical Nurse Specialists in non-palliative specialisms such as heart failure and neurological conditions. In the UK, qualified nurses’ start at Band 5 and go up to Band 8. Nurses at Band 6 have advanced training, skills and experience; those at Band 7 have managerial roles in addition

### Data collection and recording

Interviews took place between June 2017 and October 2018. The majority of participants were interviewed individually at their place of work and during work hours. However, we were receptive to workload constraints and participant preferences so undertook four interviews over the telephone, one joint interview, and three group interviews (involving three or more participants). Interviews lasted between 19 and 69 min. All participants gave written or, in the case of telephone interviews, verbal consent. An interview schedule was used as a guide allowing the flexibility to tailor and adapt each interview to the participant and their responses. All interviews were audio recorded and notes written up after the interview took place. The focus of the interviews was to explore what HCPs saw the issues to be for patients and families managing medications at home as the patient approached the end of life, and how they sought to support such patients and families in managing their medications. HCPs were asked to think of a current or recent case which had posed issues for medicines management or where this had worked well. They were then asked to situate this case within their normal practice to establish how common or rare particular issues might be. The interview guide can be found in Appendix A. All interviews were conducted by EW, GC and KP. All have considerable training and experience in sensitive topics, qualitative methods and palliative and end of life care.

### Analysis

All interviews were transcribed verbatim and fully anonymised before transcripts were uploaded to Nvivo12© and coded. Using a process of thematic analysis EW, GC and KP reviewed codes and undertook condensing and sorting. The legitimacy of categories was reviewed with the project steering group and considered in the context of the data collection methods. Several transcripts were also read and coded by our PPI co-applicant (Alan Caswell) for an additional perspective, transparency and comparison. Each transcript was coded by two team members and three were triple coded. Once coding was complete, nodes were further interrogated and refined to generate node summaries of key themes illustrated by quotations from transcripts. A number of key findings emerged during this process. These have also been compared with patient, family caregiver and bereaved family caregiver finding as part of the wider study. In June 2019 findings were presented at two workshops for HCPs (some of whom participated in the interviews) for feedback and comment. This paper focuses on the ways in which HCPs reported supporting patients and families to manage medications in the home.

## Results

The majority of HCPs in this study acknowledged that patients did not always take medications as prescribed and many reported trying to explore how to resolve this with patients and families. Dosette boxes, and more commonly ‘blister packs’, were prepared by pharmacists, and often delivered directly to patients’ homes. These were offered and perceived as a problem solving intervention to help patients to organise and remember when to take their medicines. In a number of cases these boxes could be helpful and appropriate. However, HCPs recognised that some patients and families needed more comprehensive and innovative approaches. Here we discuss the strategies reported by HCP participants, including some who described ways of thinking ‘outside the box’ in order to help support patients overcome the practical difficulties of managing their medications in the home environment. Awareness of the challenges faced by patients and family caregivers was not evident in all professional accounts as is illustrated in this insight from a community nurse in her explanation of the underpinning issues:

*It is very difficult for patients and I think, as healthcare professionals, sometimes, we can be a little bit blasé about writing prescriptions and not realising … the impact that will have [for the patient] at home when they’re faced with their twenty pills in the morning, and they’ve got to work out which one to take off, and which one to put back on, and obviously, we produce changes, they get used to the little round white one then it turns blue, and they don’t know what’s going on. So, I think it’s very important that when we’re prescribing, we spend an appropriate amount of time making sure that people understand what we’ve given them, what it’s for, what we expect it to do, but also, think about, … you’re in the home environment … you can check for the last six months’ stockpile that’s in the kitchen cupboard and things like that, to help people out. (HCP19_Community nurse).*

In general, HCPs/ HCPs tended to focus on prescribing issues, rather than express awareness or curiosity about patient perspectives and experience of medicine taking. Some seemed resigned to deliberate or inadvertent failure on the part of patients to take medication as prescribed.

*I’ve got a lady at the minute …, she’s probably on twenty three medications. You go in [to her house and look at her medication boxes] ‘oh great, they’re taking all the medication’, and then you look on the floor, and you see there’s pills all over the place. So, I don’t delude myself that any of them are taking medications as prescribed. (HCP05_Community Nurse).*

As the GP in the following quote indicates, a simple response of writing out a guide to a patient’s medication was not something they had done before nor intended to do again, despite the apparent benefits for the patient. They also note that the patient had to present to them in some considerable distress to prompt this option:

*So she came to see me in a complete sort of meltdown once, being just very upset and ... didn’t have any sort of memory about how to take her medications at all … that was just due to being very worked up I think rather than anything else going on. And I wrote down, I gave her a little sheet that said take this at this time in this dose basically, typed that out for her. And … she doesn’t want to give up her independence, it’s very, very important to her, so not being able to manage her own medications is for her a disaster … Yeah, she brought it back and showed me and said I’m using this now thank you. I’ve never done that for anyone else before. … That’s the only person I’ve ever done that for and it was only because she was so distressed by it and it is this unique set of circumstances. (HCP25_GP).*

### Effectiveness and challenges of well established solutions to medicines management

Dosettes and blister packs were often seen as an effective strategy in eliminating any confusion about what tablets to take when. HCPs viewed them as an important tool and appropriate way to support patient’s wishes to continue to manage at home independently. A few respondents observed that this system was sometimes put in place for the convenience of paid Home Care Workers enlisted to prompt patients to take medication in the home, rather than the patient. However, this could potentially undermine agency and competence, detaching patients from the process by ‘*making people further removed from taking responsibility of their own medication*’ *(HCP05_Community nurse).* They also made it difficult, if not impossible, for patients to identify individual tablets.

Dosette boxes and blister pack were recognised to pose two key issues for palliative care. One is that they do not accommodate all types of medication. Consequently, patients might have to take additional medications, such as liquids or tablets prescribed to be taken ‘as needed’, alongside the allocated pills from the box. Secondly, box contents are usually made up for a month, and cannot be easily altered if medication is changed during this period. HCPs also identified potential problems with pain medications where patients who are unaware of, or unable to identify the contents, may risk taking more pain relief than they need if they are also taking ‘as needed’ doses on top of those included in their dosette box.

*And I think actually pain does vary day to day in a patient, what they do or are not doing. And I’m very hesitant actually to put it in dosette boxes as well for that reason. And also, if you do get side effects, you get more drowsy for any other reasons, you really want to reduce this quickly and not taking the next dose and so forth. So you don’t have this variety, because in a dosette box I can’t, most of the time, see which of the pills is which, let alone the patient, and you can’t take it out and then say, ‘Ooh I think I’d better miss that because I’m feeling drowsy’. (HCP25_GP).*

Some healthcare professionals recognised the limitations of the pre-prepared dosette box for patients at the end of life and the differing needs of individual patients. Reviewing and rationalising medications was often seen as the first step to supporting effective medicines management. HCPs, especially nurses making home visits, recognised that a number of issues with medications stemmed from patients not fully understanding what their medications were, what they were for, and how they should be taken. However, they also noted despite the involvement of multiple health professionals few took responsibility for overarching management of medications and many often did not ask the right questions or investigate the underlying causes of patients’ issues with medication:

*I suppose we always think that the pharmacists are doing a lot of the explaining and a lot of the discussion … [but] I’ve never really had that conversation with a pharmacist and saying, Actually, do you just give them the Morphine, or do you tell them anything else? (HCP11_GP).*

*He was like a rabbit in the headlights, he didn’t know what to do, he’d just got all these tablets there, he didn’t know what to do, he didn’t understand what half of them were for. So his way was to not take them, … and people were going along the lines of, ‘oh those, those medicines haven’t worked, let’s try something else’, but actually, those medicines had never been taken. … And nobody had taken the time to discuss it with him. (HCP14_Specialist Palliative Care nurse).*

This was a particular issue when changes were made to medication regimes, as is common in end of life care. For these issues, taking some time to explain, and implementing a simple memory aid system to support the explanation, could be extremely effective.

*So I give [out] a lot of laminated prompt cards, that just lays out, you know,’ your aspirin is being taken for this, it should be taken with food in the morning’, and then they can follow that. And that actually helps way more than a dosette (HCP21_Pharmacist).*

HCPs also reported using items such as lockable tins for patients who could not safely manage their own medications, or where there was potential for misuse or misappropriation of medication, and dispensers with timing devices or alarms for those with impaired memory. Some reported changing the route of administration for a patient when swallowing tablets became an issue or when the number of tablets added to the burden of medicines management. One community nurse felt alternative administration routes also supported adherence as they did not hold the same negative connotations as ‘pills’ and ‘tablets’. Yet HCPs also noted that patients and families did not always know that medication came in different formulations or that they could request this.

*Actually, a lot of relatives and patients aren’t aware they can change the form of the medication and have something that’s either dispersible or in liquid form. … as I’m doing my nursing assessment, I’ll talk about medication as one of the things I’m discussing. And, so I’m saying, ‘Are you managing to swallow your medication okay?’ At that point, unless they were asked a direct question, patients don’t often flag it up to you, so, largely, ‘no’. ‘It takes me half an hour in the morning, to swallow one tablet’. And they don’t realise, actually something simple like putting it in some yogurt and having it as a bolus, might help it, help swallow it, or they won’t realise that, actually, that type of medication, you can have in a different form and have it in a syrup. (HCP06_Community nurse).*

### Thinking outside the (dosette) box

Participants described cases where they had needed to take a more innovative and active role in supporting the patient to manage medications.

*I had a gentleman who couldn’t read or write, … and he lived on his own, what we ended up doing with him, with managing his medicines, was taking one of the medicines, [sticky] taping it to a piece of paper and saying this one, and then writing next to it, like, how many times a day to take it, so three dashes …*. *you learn little ways of, often, you try something, that doesn’t work so you try something else, so it is tricky. (HCP14_Community nurse).*

In a small number of reported instances, HCPs recognised that their input was needed on a more consistent basis to support medication use. A few HCPs reported telephoning patients to remind them of recent changes to their medications. One HCP narrated an instance with a patient with memory issues who was repeatedly coming to hospital with heart failure symptoms because he was not remembering to take his medication. They subsequently rationalised this to be taken once a day and arranged for a community nurse to visit daily to prompt this administration. However, lack of time to adequately discuss and monitor medicines and related issues was acknowledge to be a limitation. This level of input was unlikely to be routinely available and the HCP who recounted this experience also noted that this was an ‘extreme’ response that could not be support for long.

Tailored solutions were particularly required in difficult and unusual situations, for example where patients or someone in their household were affected by phobias, addictions, substance abuse or the effects of dementia. One palliative care nurse described how she and her colleagues were working to find constructive and creative ways to manage pain for a patient who was determined to die at home. As the patient, his FCG and their circle of friends had issues with substance misuse, safeguards were put in place to limit the amounts of morphine they could access at any one time.

*We just introduced the patch last week, he was using a lot of the Oramorph, … he’s used the Oramorph less since the patch has gone on. … We always put dates on the bottle and the box. So we can see which bottle he’s still using and which box, and keep an eye on it as well. He knows we do that. (HCP07_Specialist Palliative Care nurse).*

This HCP also recalled a patient who expressed a fear of needles and refused any injectable medications. In the quote below the HCP described trying to balance respecting the patient’s choice and agency and providing effective care:

*We can’t use needles. So we are limited in what medication he can have to manage his symptoms anyway. We’ve got patches … And we’ve got buccal things that we can use to manage as much as we can, but obviously, it doesn’t give us the range that we would normally have. (HCP07_Specialist Palliative Care nurse).*

Recognition, and support for, patient choices about their treatment and care was a strong theme throughout the interviews. A number of HCPs described the considerable effort they were prepared to make to enable these, regardless of whether they considered them to be wise options.

*So she’d developed swallowing issues. And so we tried liquid medication, she didn’t like it. … So she went back to taking oral tablets. … And then I can think about an incident, it was interesting, at the hospice, where I got a phone call from the staff nurse saying the carers had said that she’d had a choking episode following taking tablets in the morning, and that, at the hospice, they weren’t going to be prepared to give her tablets any longer. … I said, ‘This isn’t an issue, this lady has capacity to make the decision, she doesn’t want to take liquid medication, she wants to take her tablets, the risks have been explained to her’. (HCP05_Community nurse).*

There were few references to pharmacists taking on roles to support medicines management or to other HCPs identifying the potential for greater pharmacy integration in the healthcare team.

## Discussion

Patients living at home at the end of life often have complex medication regimes with large numbers of medications, different routes of administration, and high doses of controlled drugs. In line with the international literature, the HCPs participating in this study recognised the burdens of polypharmacy at end of life [14, 16, 20–22], but did not always seem to acknowledge how the burdens of medication management added to the ‘work’ patients and FCG’s were undertaking when someone is seriously ill in the home environment [23]. FCGs are also more likely to be older, and have reduced abilities and co-morbidities of their own [24]. Until recently much of the literature on medication management focused on ‘adherence’ and ‘compliance’ and the implication for health outcomes [5, 25–28]. There is now a small but growing body of work that recognises the difficulties for patients [2], their desire to limit the amount of medications they take as far as possible [16, 29, 30] and the burdens regimes can place on FCGs when a patient is dying at home [19, 21, 31]. Indeed, the international literature indicates that in other countries, particularly the United States and Australia, FCGs are regularly undertaking even more advanced tasks in administering sub-cutaneous medications prescribed for end of life symptoms [32–36]. While there have been some moves towards this in the UK the feasibility of this approach is still being assessed [37, 38].

This paper has explored ways in which HCPs in a UK study reported on well established solutions to medicines management and more innovative ‘outside the box’ thinking in order to support patients and families with medication needs. Some expressed resignation that patients often failed to, could not or did not want to take their medications as prescribed. Not all HCPs recognise that a strategy of verbal explanation on its own was not always effective in helping patients to understand and manage their medications, particularly at the end of life. Accounts, particularly from specialist palliative care nurses, noted that when multiple health professionals were involved there was little coordination or designated responsibility for medications management. HCPs in this study often focused on the prescription element of the process, aiming to ensure appropriate symptom control by undertaking simple changes to regimes and routes of administration. However, while these may present as obvious and standard initial options not all HCPs in the study thought to implement them. These adaptations often made a considerable difference to patients’ ability to manage their medicines effectively. In other instances, HCPs reported having to be quite innovative, and explore a number of routes to help patients overcome the *practical* problems involved in organising and taking their medicines effectively.

While not aimed specifically at palliative care, current UK guidance [17, 39] advocates for professionals to take account of individual patient needs, preferences for treatment, health priorities and lifestyle and recognise that ‘medicines are likely to be just one aspect of a person’s care’ [39]. However, to date there has been little work to explore ways of supporting patients and families in medicines management in order to make their lives less stressful [4, 40].

The ability to maintain accurate medicine taking could be a critical factor in avoiding unscheduled hospital admissions and determining whether a patient could remain living at home [41–43]. This paper seeks to extend beyond the compliance/adherence discourse [44] to recognise the reasonable difficulties patients and families often face in managing complex regimes and how HCPs play a proactive role in helping them overcome these [5]. HCPs in this study often reported encouraging the use of simple dose administration aids such as dosette boxes and blister packs. These can be extremely useful and can support patients to remain at home by helping them to manage their own medication. They can also be a useful tool in supporting Home Care Workers to safely prompt and administer medications in patients’ homes [7–9, 45]. Conversely, these aids can have negative implications for a person’s independence and agency when they disempower patients by removing responsibility for, and understanding of, their own medications [46]. They also have practical disadvantages when, as is common in end of life care, medications need to be changed rapidly and frequently [7]. This increases risk of adverse events, lack of effective symptom management, and instability of some medications when stored outside their original packs. These types of issues add to the challenge of optimising pain control in the home environment [47, 48].

This paper has demonstrated that there is scope for much greater understanding of the reality of patient and FCG experience, the difficulties they face, and the potential for HCPs to engage with innovative and tailored ways of supporting medicines management for seriously ill patients being cared for and dying at home. Our findings further echo those of current literature in that the majority of participants rarely recognised the current or potential contribution of pharmacists as part of the palliative care team [49–54].

### Limitations

A strength of the study is that it represents the views of a wide range of HCPs, including pharmacists, working in diverse community healthcare services and geographic locations. However, the study location in two adjacent English counties means that the findings may not be typical of other settings. It is also important to note that 17/40 participants were considered to be specialists in palliative care and 4/40 condition specific specialists. As such, we would expect them to manage a more complex caseload and be able to implement a wider range of ways to support patients in managing their medications. We recognise that as self-selected participants, they are likely to have an interest and specialist expertise in the management of medications. Interviews may involve accounts in which respondents are likely to be motivated to give a good account of their practice. We are unable to know how this compares with what they actually do in practice. However, data from patients and FCGs in other parts of the study suggest that proactivity by HCPs is far from routine and especially in the last weeks and days of life when families are confronted with real anxieties about the management of medications.

## Conclusions

If we move the discourse forward from one of compliance and adherence and recognise the reasonable, and often practical, difficulties patients and FCG face when managing medications we can look at ways in which HCPs can help them to overcome these issues. Medication reviews, reduction of problematic polypharmacy and the use of dose administration aids such as dosette boxes are some of the established ways in which HCPs can support patients and families in medicines management at the end of life. However, there may be times when HCPs need to ‘think outside the box’ in order to identify and support patients to safely, effectively and independently manage their medications.

## Supplementary information

**Additional file 1.** WP1 HCP Interview Topic Guide

## Data Availability

The datasets generated and/or analysed during the current study are not publicly available as they are personal accounts of patients’, families’ and healthcare professional’s experiences. It is essential that we maintain confidentiality and anonymity but sufficiently anonymised parts of the data are available from the corresponding author on reasonable request.

## Abbreviations

FCG: Family caregiver
HCP: Healthcare professional
