# Supplementary material for: An exploration of the experiences of professionals supporting patients approaching the end of life in medicines management at home. A qualitative study
Source: BMC Palliat Care. 2020 May 11;19:66. doi: 10.1186/s12904-020-0537-z (PMC7216477; doi:10.1186/s12904-020-0537-z)
Supplement: Supplementary file 1 — Additional file 1. WP1 HCP Interview Topic Guide [file 12904_2020_537_MOESM1_ESM.docx]

**WP1 HCP Interview Topic Guide**

1. **Introduction**
   - *Introduction to researcher and study*
   - *Focus on medicines management especially among patients/families*
     - - *with illness other than cancer*
       - *from BAME and other minority and underserved populations*
   - *informal discussion; confidential; can stop at any time; no pressure to answer questions/discuss specific topics*
   - *permission to tape*
   - *Completion of consent to interview*
2. Can you tell me about a specific case (or cases) from your current/recent case load and your involvement in supporting the patient and family to manage their medicines at home during the last months of life?
   - *If possible from BAME/underserved population and/or not affected by cancer*
   - *relationship with patient/family; involvement in care and support*
   - *main issues re illness and treatment*
   - *other services/HPs Respondent liaises with in providing care*
     - *specifically re managing medicines*
   - *patient and family coping*
     - *Understanding of prognosis*
     - *Understanding of treatment*
     - *Compliance*
   - *communication/exchange of information*
     - *with patient and family*
       - *who is involved, what role do they play:*
         - *making decisions*
         - *access and administration*
         - *administration*
         - *storage*
     - *with other HCPs and services*
       - *who is involved, what role do they play*
       - *sources of support/advice*
       - *sources of difficulty/tension*
   - *prescribing/use of AMs*
     - *Professional responsibility*
   - *issues/concerns regarding current treatment and medicines management in this case*
   - *anticipation of what will happen/issues arising in next few months?*
   - *How typical/unusual do you consider this case? (Why)*
3. Thinking more generally, I’d like to get a picture of the healthcare professionals and services with which you interact in supporting patients and families to manage medicines.
   - *Construct ecogram: using blank sheet of paper ask R to represent his/her network of professional contacts and services*
   - *Establish – range and frequency of contacts; professional roles and allocation of responsibility; interface between community/primary services and secondary care; involvement and support for patients and families; prescribing and use of AMs.*
   - *Areas/sources of support*
   - *Assessment of how will the network functions/how it could be improved*
     - *Any scope for greater/more effective involvement of CPs/pharmacy services?*
4. What do you consider to be the main issues and difficulties experienced by patients and families in managing medicines at home?
   - *Re access, administration, coordination, storage, concerns about symptom control, side effects, responsibility, complexity, compliance etc.*
   - *Sources of support, advice and information*
   - *How is the decision to prescribe and use AMs made and communicated to patients and families?*
     - *Engagement in discussion and decisions*
5. What do you think of the support with medicines management currently available to patients and families? Any differences
   - *between illnesses*
   - *within BAME and underserved groups*
   - *between services/resulting from referrals*
   - *between locations*
6. Are there any ways you feel the system could be improved: How?
   - *And specifically for BAME and underserved groups*
   - *For patients with illnesses other than cancer*
   - *Greater input from CPs?*
7. How confident do you feel in supporting patients and families to manage medicines at home?
   - *Knowledge, experience, training*
   - *Additional training, resources, support needed*
     - *For self and HCPs*
     - *For patient and family*
   - *Concerns and how they might be resolved*
8. Conclusion
   - *Anything else, missed, not discussed?*
   - *Confirm personal details as required:*

*Age, professional role and position, length in current post, experience, specialist training in palliative and EOLC, AMs.*

- Thanks!
